# The relationship of alpha-synuclein to mitochondrial dynamics and quality control

**DOI:** 10.3389/fnmol.2022.947191

**Published:** 2022-08-26

**Authors:** Naomi J. Thorne, David A. Tumbarello

**Affiliations:** Biological Sciences, University of Southampton, Southampton, United Kingdom

**Keywords:** mitochondria, Parkinson’s disease, mitochondrial quality control, vesicle transport, lysosome, membrane trafficking

## Abstract

Maintenance of mitochondrial health is essential for neuronal survival and relies upon dynamic changes in the mitochondrial network and effective mitochondrial quality control mechanisms including the mitochondrial-derived vesicle pathway and mitophagy. Mitochondrial dysfunction has been implicated in driving the pathology of several neurodegenerative diseases, including Parkinson’s disease (PD) where dopaminergic neurons in the substantia nigra are selectively degenerated. In addition, many genes with PD-associated mutations have defined functions in organelle quality control, indicating that dysregulation in mitochondrial quality control may represent a key element of pathology. The most well-characterized aspect of PD pathology relates to alpha-synuclein; an aggregation-prone protein that forms intracellular Lewy-body inclusions. Details of how alpha-synuclein exerts its toxicity in PD is not completely known, however, dysfunctional mitochondria have been observed in both PD patients and models of alpha-synuclein pathology. Accordingly, an association between alpha-synuclein and mitochondrial function has been established. This relates to alpha-synuclein’s role in mitochondrial transport, dynamics, and quality control. Despite these relationships, there is limited research defining the direct mechanisms linking alpha-synuclein to mitochondrial dynamics and quality control. In this review, we will discuss the current literature addressing this association and provide insight into the proposed mechanisms promoting these functional relationships. We will also consider some of the alternative mechanisms linking alpha-synuclein with mitochondrial dynamics and speculate what the relationship between alpha-synuclein and mitochondria might mean both physiologically and in relation to PD.

## Introduction

Mitochondria are dynamic organelles that are required for survival in all eukaryotic cells. Initially evolved from the engulfment of proteobacteria by the ancestors of modern eukaryotes, mitochondria possess their own DNA (mtDNA), encoding machinery to enable energy production in the form of ATP (Lane and Martin, 2010). Mitochondria have essential roles in cell homeostasis and health, not least because the ATP they generate fuels all biochemical and metabolic reactions. However, beyond their role in ATP production mitochondria are known to be essential regulators of calcium balance, phospholipid transfer, and apoptosis (Dunchen, 2000; Jeong and Seol, 2008; Kojima et al., 2016). They also have extensive functional contacts with many cellular organelles including the endoplasmic reticulum, peroxisomes, lipid droplets and lysosomes, which are essential for lipid exchange and the regulation of signaling processes associated with the immune response, autophagy and apoptosis (Tait and Green, 2012; Breda et al., 2019). Consequently, mitochondrial dysfunction is severely detrimental to the proper function of an organism, resulting in a series of toxic signaling pathways that impact cell survival.

## Mitochondrial quality control

Mitochondrial dysfunction has long been associated with the aging process. During aging, both respiratory capacity and ATP production are reduced in mitochondria, which is coupled to an increase in the production of reactive oxygen species (ROS) (Conley et al., 2000; Capel et al., 2005; Santanasto et al., 2015). A by-product of oxidative phosphorylation during ATP production, ROS are considered both beneficial and destructive to cell health; essential regulators of defensive signaling pathways physiologically, but instigators of oxidative damage when in excess (Bardaweel et al., 2018). In the innate immune system, ROS promote the release of pro-inflammatory cytokines in response to both damage-associated molecular patterns (DAMPs) and pathogen-associated molecular patterns (PAMPs) as well as co-ordinating the activation and assembly of inflammasomes (Yang et al., 2013; Bardaweel et al., 2018). ROS also have an important role in the induction of autophagy through modification of proteins such as Atg4, stimulating recruitment of LC3 to autophagosomal membranes (Scherz-Shouval et al., 2007). However, excessive levels of ROS can alter mitochondrial membrane permeability, damage protein complexes and contribute to the accumulation of mtDNA mutations, resulting in dysfunctional electron transfer and disrupted mitochondrial function (Guo et al., 2013; Zorov et al., 2014; Sun et al., 2016; Jang et al., 2018). As such, ROS levels must be finely balanced to restrict oxidative damage and preserve mitochondrial health, particularly during aging. Alongside increased ROS production, a reduction in protein degradation has been shown with age, contributing to the presence of damaging misfolded, unfolded or oxidized mitochondrial proteins (Cadenas and Davies, 2000; Bakala et al., 2003). Since mitochondrial dysfunction is intrinsically linked to cell survival, age-associated mitochondrial damage can result in cell death. Consequently, dysfunctional mitochondria have been associated with the pathogenesis of several neurodegenerative diseases, including Alzheimer’s disease (AD), Parkinson’s disease (PD), Huntington’s disease (HD), and amyotrophic lateral sclerosis (ALS) (Beal, 2005; Reddy and Beal, 2008; Muyderman and Chen, 2013; Franco-Iborra et al., 2018).

In post-mitotic cells such as neurons which have an average mitochondrial half-life of 20–25 days, mitochondria are continuously being created, removed and modified (Menzies and Gold, 1971). Constant remodeling of the mitochondrial network occurs by the action of dynamin-like GTPases, which regulate mitochondrial dynamics through fission and fusion events (Yapa et al., 2021). Mitochondrial fusion enables the unification of two individual mitochondria into a single organelle, facilitating complementation of dysfunctional mitochondria and improving oxidative capacity (Youle and van der Bliek, 2012). Fusion occurs by mechanistically distinct processes at the outer mitochondrial membrane (OMM) and inner mitochondrial membrane (IMM), with OMM fusion regulated by Mfn1 and Mfn2 and IMM fusion co-ordinated by Opa1 (Meeusen et al., 2004). Fission is defined by the splitting of one mitochondrion into two daughter organelles which is coordinated by dynamin-1-like protein (Drp1); proposed to act by forming a constricting ring around the organelle which severs the OMM (Youle and van der Bliek, 2012). This limits the extent of any mitochondrial damage, allowing one organelle to remain fully operative while the other contains damaged components, which can be degraded by mitochondrial quality control (mito-QC) mechanisms. A healthy mitochondrial population results from effective fission and fusion combined with mito-QC systems; features mitochondria have retained from their ancestors that protects against damage through self-surveillance and defense mechanisms. Though active at a basal level, mito-QC processes can be upregulated in response to oxidative stress (Eisner et al., 2018; Roca-Portoles and Tait, 2021). Distinct pathways are employed depending on the extent of insult, but all endeavor to intervene and restrict mitochondrial damage to avoid toxicity and cell death.

Since mitochondria are the primary producers of ROS in the cell, they possess various antioxidant enzymes which act as a first line of defense against oxidative stress (Brillo et al., 2021). These include superoxide dismutase, catalase and glutathione peroxidase which scavenge excess ROS and catalyze their conversion into less reactive and damaging species (Matés et al., 1999; Yang and Lian, 2020). As well as removing mitochondrial ROS, these enzymes provide mitochondria with a redox buffering ability that allows quenching of cytosolic hydrogen peroxide to ease cellular oxidative stress (Ježek and Hlavatá, 2005; Mailloux, 2018). When ROS production exceeds antioxidant enzyme capability, damaged lipids and proteins accumulate within the mitochondria, impacting their efficient function. At this point, mito-QC systems can be employed at the molecular level, working to ensure the correct stoichiometry and folding of mitochondrial proteins to limit damage (Jin and Youle, 2013). Individual unfolded, misfolded or oxidized soluble proteins can be digested by mitochondrial chaperones or proteases within the organelle and their transcription can be upregulated following initiation of the mitochondrial unfolded protein response (mtUPR) (Jin and Youle, 2013). Damaged proteins on the OMM are targeted by degradation pathways in the cytoplasm; primarily through the ubiquitin-proteasome system (UPS) (Taylor and Rutter, 2011). The collective action of the ubiquitin conjugation machinery tags these proteins with ubiquitin, enabling their recognition by ubiquitin-binding proteins and their subsequent delivery to the 26S proteasome where they are degraded (Taylor and Rutter, 2011; Xu and Jaffrey, 2011).

The next level of mito-QC is conducted by mitochondrial-derived vesicles (MDVs); vesicular compartments formed when small regions of the mitochondrial membrane bud off from the mitochondria (Sugiura et al., 2014). These structures contain locally damaged mitochondrial components, although recently they were shown to turnover fully assembled protein complexes (Sugiura et al., 2014; König et al., 2021). Once formed, MDVs are then trafficked away from the mitochondrion and fuse with the lysosome, allowing selective removal of damaged proteins and lipids, while preserving the remainder of the organelle (McLelland et al., 2014). Recent evidence has shown that MDV biogenesis relies on the activity of GTPases essential for mitochondrial dynamics (König et al., 2021). The current model suggests that protrusion of mitochondrial membrane buds is mediated by microtubule-dependent pulling by Miro1/2, while Drp1-dependent scission cleaves membranes to leave an independent vesicle (König et al., 2021). MDVs exist as one of two structural subtypes: single-membraned vesicles containing only OMM proteins, or double-membraned vesicles that additionally incorporate proteins from the IMM and matrix (Neuspiel et al., 2008; Soubannier et al., 2012a,b). As such, MDVs exhibit cargo selectivity and traffic to different end destinations dependent on the cargo they carry. For example, double-membraned MDVs containing the OMM protein MAPL shuttle to peroxisomes whereas those containing the IMM protein PDH are trafficked to the lysosome for degradation along with single-membraned MDVs positive for the OMM protein TOM20 (Neuspiel et al., 2008; Soubannier et al., 2012a,b). Evidence indicates that these latter classes of MDVs are upregulated in response to oxidative stress and can selectively incorporate oxidized cargo, providing a mechanism by which mitochondria can sequester and exclude damaged proteins to retain organelle functionality under stress conditions (Soubannier et al., 2012b).

Upon excessive oxidative stress, mitochondria experience alterations in membrane permeability, promoting formation of the mitochondrial permeability transition pore (Stewart and Heales, 2003; Guo et al., 2013). The resultant changes in ion balance induce loss of mitochondrial membrane potential, triggering degradation of the whole organelle through the mitochondrial autophagy pathway, known as mitophagy (Yang and Klionsky, 2010). Preceded by mitochondrial fission, mitophagy occurs when the less functional of the two daughter organelles is deemed to be damaged beyond repair (Twig et al., 2008). This suggests the existence of a mitochondrial damage threshold, above which lower-level repair mechanisms such as the MDV pathway are no longer sufficient defense mechanisms. Such a threshold has not yet been defined, but is likely to be influenced by both the integrity of membrane potential and the extent of oxidative damage (McLelland et al., 2014). The most well characterized stress-induced mitophagy pathway is regulated by PINK1 and Parkin, although PINK1/Parkin-independent mitophagy pathways also exist (Allen et al., 2013; Ke, 2020). PINK1 is a mitochondrial-targeted serine/threonine kinase that is rapidly imported across the mitochondrial membrane and degraded under physiological conditions, but accumulates on the OMM upon mitochondrial depolarization leading to the recruitment and activation of the E3 ubiquitin ligase, Parkin, which facilitates the ubiquitylation of OMM proteins (Narendra et al., 2010; Rüb et al., 2017). A linkage between the damaged, ubiquitylated mitochondrion and autophagosomal membrane is mediated by autophagy receptors such as NDP52, optineurin and TAX1BP1 which directly associate with both polyubiquitin and the LC3 family of autophagosome membrane proteins (Lazarou et al., 2015; Ryan and Tumbarello, 2018). This allows the cargo to be tethered to the autophagosomal membrane, mediating engulfment of the mitochondrion by the autophagosome and subsequent degradation by the endolysosomal system (Stolz et al., 2014). Removal of the whole organelle by mitophagy prevents the expansion of damage to the rest of the cell and is reserved for inordinate levels of stress due to the high energy demand it requires both to co-ordinate mitophagy and to replace the eliminated mitochondria (Cadete et al., 2016).

## Mitochondrial quality control in Parkinson’s disease

Mito-QC mechanisms are essential for the preservation of mitochondrial integrity, particularly in aging and disease. Accordingly, the effective functioning of these processes is paramount to ensure cell survival. There is significant evidence linking dysregulation of mito-QC to the pathology of several neurodegenerative diseases, but the relationship is notably strong between mito-QC and PD (Wang et al., 2009; McCoy and Cookson, 2012; Guedes-Dias et al., 2016; Gao and Zhang, 2018). A progressive and debilitating movement disorder, PD affects 1% of people over 60 years old and currently has no cure and limited treatment options to improve quality of life (De Lau and Breteler, 2006). In the brain, selective degeneration of dopaminergic neurons in the substantia nigra pars compacta (SNpc) is responsible for a myriad of cognitive and psychiatric symptoms on top of motor difficulties that characterize the disease (Dauer and Przedborski, 2003). The precise mechanism of neuronal death in PD is still not understood, but mitochondrial dysfunction has been identified as a potential basis for targeted cell degeneration (Martin et al., 2006; Ganjam et al., 2019). SNpc neurons are particularly vulnerable to oxidative stress due to their high metabolic burden, evoked by their copious synaptic connections and the elevated production of ROS as a result of their intrinsic dopamine metabolism (Pacelli et al., 2015). The already delicate energy balance in these neurons indicates they are especially sensitive to mitochondrial dysfunction, thus requiring precise damage control by mito-QC to avoid further exacerbation of mitochondrial stress and resultant neuronal degeneration.

The evidence for mitochondrial dysfunction as a driver of PD pathology was first highlighted when synthetic heroin drug users inadvertently ingested the mitochondrial inhibitor, MPTP (1-methyl-4-phenyl-1,2,3,6-tetrahydropyridine), resulting in symptoms with striking comparability to PD (Davis et al., 1979). Post-mortem assessment revealed the degeneration of SNpc neurons and the presence of Lewy bodies, which are intracellular inclusions that are a hallmark characteristic of PD pathology (Davis et al., 1979; Langston et al., 1983). Alongside MPTP, other chemical inhibitors of mitochondrial function such as 6-hydroxydopamine (6-OHDA) and rotenone are now used to generate animal models of PD (Ungerstedt, 1971; Betarbet et al., 2000). More recently, genome-wide association studies (GWAS) have validated the relationship between mitochondria and PD pathology following the identification of numerous PD-associated genetic perturbations (Chang et al., 2017; Billingsley et al., 2019). Two of the first gene mutations to be linked with familial PD pathogenesis were in the mitophagy regulators PINK1 and Parkin (Kitada et al., 1998; Valente et al., 2004). Interestingly, many of the mutations linked to PD are in genes with defined roles in mito-QC pathways, including direct associations with mitochondria and connections with the downstream endolysosomal compartment (Table 1). The existence of these provides support for the hypothesis that dysfunction in mito-QC is intrinsically linked to PD pathology. Aside from these, several PD-associated mutations have been found within the SNCA gene which codes for alpha-synuclein (Polymeropoulos et al., 1997; Siddiqui et al., 2016).

**TABLE 1:** Mitochondrial and endolysosomal associated genes linked to Parkinson’s disease.

Gene	Protein	Function	References
ASAH1	*N*-acylsphingosine amidohydrolase 1	Lysosomal lipid hydrolase	Aharon-Peretz et al., 2004; Robak et al., 2017
ATP13A2	ATPase cation transporting 13A2	Late endosomal transporter and lysosomal polyamine exporter	Ramirez et al., 2006
ATP6V0A1	ATPase H + transporting V0 subunit a1	Proton transporter regulating organelle acidification	Morel, 2003; Chang et al., 2017
CHCHD2	Coiled-coil-helix-coiled-coil-helix domain-containing protein 2	Localized to mitochondria intermembrane space; associated with the biogenesis and regulation of ETC proteins	Funayama et al., 2015; Kee et al., 2021
COQ7	Coenzyme Q7 hydroxylase	Mitochondrial enzyme required for coenzyme Q synthesis	Freyer et al., 2015; Chang et al., 2017
CTSB	Cathepsin B	Lysosomal protease required for autophagy cargo degradation	Chang et al., 2017; Yadati et al., 2020
CTSD	Cathepsin D	Lysosomal endopeptidase	Benes et al., 2008; Robak et al., 2017
PARK7	Parkinsonism associated deglycase (DJ1)	Redox-sensitive chaperone and protease	Bonifati et al., 2003; Hijioka et al., 2017
FBXO7	F-box only protein 7	Component of the SCF E3 ubiquitin ligase complex; role in PINK1-Parkin mitophagy	Fonzo et al., 2009; Burchell et al., 2013
GALC	Galactosylceramidase	Lysosomal hydrolase	Chang et al., 2017; Robak et al., 2017
GBA	Glucosylceramidase Beta	Lysosomal hydrolase	Sidransky et al., 2009; Magalhaes et al., 2016
LRRK2	Leucine rich repeat kinase 2	Serine/threonine kinase regulating Rab GTPase function in the endolysosomal system	Paisán-Ruíz et al., 2004; Bonet-Ponce et al., 2020
PRKN	Parkin RBR E3 ubiquitin protein ligase	Ubiquitylates mitochondrial proteins and an essential mitophagy regulator	Kitada et al., 1998; Pickrell and Youle, 2015
PINK1	PTEN induced kinase 1	Mitochondrial damage sensor; recruits and activates Parkin to initiate mitophagy	Valente et al., 2004; Pickrell and Youle, 2015
RAB7L1	RAB7, member RAS oncogene family-like 1	Recruits LRRK2 to the Golgi to promote Golgi-derived vesicle formation	Beilina et al., 2014; Nalls et al., 2014
SCARB2	Scavenger receptor class B member 2	Endosomal and lysosomal membrane protein associated with lipid transport and GBA targeting	Do et al., 2011; Gonzalez et al., 2014
SMPD1	Sphingomyelin phosphodiesterase 1	Lysosomal lipid hydrolase	Schuchman, 2010; Alcalay et al., 2019
TMEM175	Transmembrane protein 175	Potassium channel in late endosomes and lysosomes	Nalls et al., 2014; Zhang et al., 2020
VPS35	VPS35 retromer complex component	Subunit of the retromer complex required for endosomal retrograde transport	Zimprich et al., 2011; Deng et al., 2013

ETC, Electron Transport chain; SCF, SKP1-CUL1-F-box protein.

## Alpha-synuclein

The most well-researched aspect of PD pathology relates to alpha-synuclein; a small, 140 amino acid protein that has both physiological and pathogenic roles in the cell. Alpha-synuclein is comprised of three domains: an N-terminus that mediates membrane binding, a NAC domain which is responsible for protein aggregation and a C-terminus that binds calcium to increase its lipid-binding capacity (Emamzadeh, 2016; Lautenschläger et al., 2018). Able to exist in several conformations, the dynamic structure of alpha-synuclein varies depending on its cellular location (Ahn et al., 2002; Emamzadeh, 2016). Alpha-synuclein is hypothesized to exist as an unfolded monomer physiologically, shuttling between residing in the cytoplasm and binding to highly curved phospholipid membranes, upon which it adopts an amphipathic helical structure (Bartels et al., 2011; Burré et al., 2013; Emamzadeh, 2016). Investigations into the membrane binding capability of alpha-synuclein using NMR revealed that it has high affinity for unsaturated lipids with small anionic head groups and polyunsaturated acyl chains (Wang et al., 2010; Pfefferkorn et al., 2012). Accordingly, alpha-synuclein preferentially binds to highly curved membrane structures such as synaptic vesicles (Drin and Antonny, 2010; Emamzadeh, 2016). Due to this affinity and its enrichment at pre-synaptic terminals, alpha-synuclein has mostly been characterized as a synaptic protein; sensing membrane curvature and regulating vesicle trafficking, recycling and release close to the plasma membrane through the assembly of SNARE complexes (Middleton and Rhoades, 2010; Burré et al., 2014). Alpha-synuclein’s function away from the synapse is poorly understood, though recent evidence has indicated a localization elsewhere in the cell, including in the nucleus and at mitochondrial membranes (Maroteaux et al., 1988; Nakamura et al., 2008).

Most research in the field of PD has focused on pathological forms of alpha-synuclein. As an aggregation-prone protein, alpha-synuclein can recruit other monomers to produce oligomers, protofilaments and fibrils which eventually form Lewy body inclusions (Cremades and Dobson, 2018). Although evidence has shown that both oligomeric and fibrillar alpha-synuclein exert harmful effects on the cell, fibrillar alpha-synuclein is more stable and is suggested to be less damaging (Winner et al., 2011). One of the ways that fibrils induce harm is by the release of pre-fibrillar oligomeric species, hypothesized to be primarily responsible for alpha-synuclein-induced neuronal toxicity (Lashuel et al., 2013). The transient nature of oligomeric alpha-synuclein has meant their cellular consequences have been difficult to determine, and the precise mechanism that promotes cell death is unclear (Lööv et al., 2016; Hijaz and Volpicelli-Daley, 2020). Animal models of PD using exogenously delivered alpha-synuclein aggregates or alpha-synuclein overexpression exhibit a toxic gain-of-function phenotype in a range of cellular systems (Lööv et al., 2016; Hijaz and Volpicelli-Daley, 2020). This includes disruption to neurotransmitter release, intracellular trafficking and protein degradation amongst other processes, having a global impact on the cell that results in degeneration (Cooper et al., 2006; Sousa et al., 2009; Nemani et al., 2010). Of note, alpha-synuclein oligomers have been shown to exert toxicity at mitochondria and throughout the endolysosomal system (Melo et al., 2018; Teixeira et al., 2021). Alpha-synuclein aggregates preferentially bind to mitochondria, not only reducing ATP production but also inducing fragmentation with subsequent impacts on mitophagy (Liu et al., 2009; Choubey et al., 2011; Wang et al., 2019).

Additionally, alpha-synuclein can impact protein degradation. Like many aggregate-prone proteins, alpha-synuclein itself is primarily removed from the cell by the lysosome (Mak et al., 2010). Alpha-synuclein aggregates have been shown to impair lysosomal function, potentially through depletion of digestive enzymes such as glucocerebrosidase (GCase) and cathepsin D (Cuervo et al., 2004; Mazzulli et al., 2011; Hoffmann et al., 2019). The presence of aggregated alpha-synuclein species in lysosomes thus creates a feedback loop that further potentiates alpha-synuclein pathology. Endogenous alpha-synuclein is also targeted by chaperone-mediated autophagy (CMA) where its lysosomal translocation is facilitated by an interaction with LAMP2A (Cuervo et al., 2004). Interestingly, data indicates that although mutant forms of alpha-synuclein still bind to LAMP2A, they appear to have a universal inhibitory effect on CMA by blocking receptor function (Cuervo et al., 2004). As such, mutant alpha-synuclein not only reduces its own degradation, but that of other long-lived cellular proteins that are CMA substrates.

Alpha-synuclein’s capacity to influence both mitochondrial and endolysosomal function pathologically suggests a significant impact for alpha-synuclein oligomers on mito-QC (Melo et al., 2018; Teixeira et al., 2021). Whether this could represent a key mechanism behind alpha-synuclein-induced cellular degeneration is an important question to be addressed. Given that many genes carrying PD-associated mutations are involved in mito-QC pathways and SNpc neurons are especially sensitive to mitochondrial dysfunction, a potential explanation for selective SNpc neuron death could be an alpha-synuclein-induced impact on efficient mito-QC function. Although dysregulation in mito-QC and the presence of alpha-synuclein aggregates are fundamental elements of PD pathology, limited work has investigated the relationship between these two aspects (Henderson et al., 2019; Eldeeb et al., 2022). Support for a functional connection between alpha-synuclein and mito-QC also comes from a physiological context. The ability of monomeric alpha-synuclein to associate with mitochondria and endomembrane structures could reflect a non-pathological role for alpha-synuclein within the mito-QC network (Ellis et al., 2005; Ludtmann et al., 2016). However, there is a considerable lack of research into defining alpha-synuclein’s function in this context outside of a pathological environment. In addition, much of the current literature addressing the relationship between alpha-synuclein and mitochondria is conflicting, providing a limited consensus on the influence of alpha-synuclein on mitochondrial function, dynamics and quality control both physiologically and in PD.

## Alpha-synuclein association with mitochondria

Several studies have significantly improved our understanding of alpha-synuclein’s association with mitochondria (Ellis et al., 2005; Devi et al., 2008; Chinta et al., 2010; Ludtmann et al., 2016). In the substantia nigra of PD patients, pathogenic alpha-synuclein accumulation was shown to be coupled to a dramatic increase in its localization to mitochondria (Devi et al., 2008). Further investigation in human dopaminergic neurons revealed that alpha-synuclein was imported into the mitochondria and was associated with the IMM (Devi et al., 2008). This selective localization was supported by the identification of a cryptic mitochondrial-targeting sequence in the N-terminus of alpha-synuclein (Devi et al., 2008). The potential for this was hinted earlier due to alpha-synuclein’s ability to form an amphipathic helix; a common feature of many mitochondrial-targeted proteins (Von Heijne, 1986; Davidson et al., 1998). Progressive amino acid deletion from the N-terminus indicated the presence of a 32-amino acid region, containing the mitochondrial-targeting sequence, required for alpha-synuclein’s localization to mitochondria (Devi et al., 2008). The existence of this signal alludes to a potential physiological function for alpha-synuclein in mitochondria. Supporting this suggestion, alpha-synuclein has been shown to interact with and modulate ATP synthase, with a reduction in both ATP synthase and complex I activity reported in alpha-synuclein knockout mice (Ellis et al., 2005; Ludtmann et al., 2016). These studies indicate that endogenous alpha-synuclein may play a regulatory role for the function of essential proteins in the respiratory chain. Interestingly, complex I impairment has also been observed upon alpha-synuclein overexpression, suggesting an excess of alpha-synuclein could interfere with its physiological role, perhaps due to oligomer formation (Devi et al., 2008; Chinta et al., 2010). It should be noted there is also evidence which does not support a physiological association of alpha-synuclein with mitochondria, indicated by minimal monomeric alpha-synuclein association with isolated neuronal mitochondria (Wang et al., 2019). However, it must be considered that this study applied alpha-synuclein monomers exogenously, which may not mimic the behavior of endogenous alpha-synuclein (Wang et al., 2019).

There is considerable variation in reports evaluating the localization of alpha-synuclein to mitochondrial membranes. Several studies have shown an enrichment of alpha-synuclein at the IMM under pathological conditions, including in PD brain, which has been linked to its interaction with the mitochondrial-specific phospholipid, cardiolipin (Devi et al., 2008; Nakamura et al., 2011; Robotta et al., 2014). Enriched at the IMM, cardiolipin can directly bind to alpha-synuclein monomers and facilitate their assembly into helical structures. Exogenous delivery of oligomeric alpha-synuclein induced formation of membrane pores, mitochondrial swelling and cytochrome C release which was dependent on the presence of cardiolipin, suggesting a functional relationship at the IMM (Camilleri et al., 2013; Ghio et al., 2019). It has been suggested that formation of such pores is due to the insertion of annular alpha-synuclein protofibrils into phospholipid membranes like the IMM, generating pore-like structures that directly influence membrane permeability (Ding et al., 2002; Tsigelny et al., 2012). The presence of alpha-synuclein at the IMM raises questions about how it is being imported into the mitochondria. In a mammalian cell model, alpha-synuclein import was shown to be dependent on an intact mitochondrial membrane potential and was blocked upon inhibition of ATP synthase (Devi et al., 2008). More specifically, alpha-synuclein import was halted by inhibition of TOM40, a key subunit that forms part of the TOM import complex (Devi et al., 2008). The TOM complex facilitates the mitochondrial entry of most mitochondrial-targeted precursor proteins and FRET analysis has confirmed co-localization with the TOM20 subunit, supporting the role for this complex in mediating alpha-synuclein import (Harner et al., 2011; Martínez et al., 2018). Interestingly, though low levels of alpha-synuclein could be washed out from mitochondria, high levels could not, suggesting that mitochondrial internalization of alpha-synuclein becomes irreversible at high concentrations (Martínez et al., 2018). This response could be related to protein conformation, since the aggregation propensity of alpha-synuclein increases with protein concentration (Afitska et al., 2019). As such, the potential formation of alpha-synuclein oligomers inside mitochondria could pose a danger to mitochondrial health.

Oligomeric alpha-synuclein species also localize to the OMM and directly bind the TOM20 subunit (Di Maio et al., 2016; Valdinocci et al., 2021). Since the TOM complex is essential for protein import, it not only provides a mechanism for alpha-synuclein internalization but also represents a potential site of pathological damage. Crucially, post-translationally modified alpha-synuclein species, such as dopamine-modified and S129-phosphorylated, prevent TOM20 from interacting with its co-receptor TOM22, blocking TOM-mediated mitochondrial protein import (Di Maio et al., 2016). Other neurodegenerative disease-linked proteins such as amyloid-precursor protein (APP) have also been shown to impede protein import following accumulation on the OMM and obstruction of the TOM40 subunit, behaving similarly to alpha-synuclein (Gottschalk et al., 2014). Interestingly, the TOM40 subunit has been shown to be altered in mouse models of PD, with TOM40 overexpression rescuing alpha-synuclein-induced toxicity (Bender et al., 2013). It can be inferred that oligomeric alpha-synuclein may negatively influence protein import when bound to the OMM or directly to the TOM complex, resulting in notable effects on mitochondrial health. It has also been suggested that alpha-synuclein binds to mitochondrial-associated endoplasmic reticulum (ER) membranes (MAMs) (Guardia-Laguarta et al., 2014; Paillusson et al., 2017). Pathogenic overexpression of alpha-synuclein disrupts calcium exchange between the ER and mitochondria, resulting in perturbations in ATP production (Paillusson et al., 2017).

## Alpha-synuclein influence on mitochondrial dynamics

**FIGURE 1:**
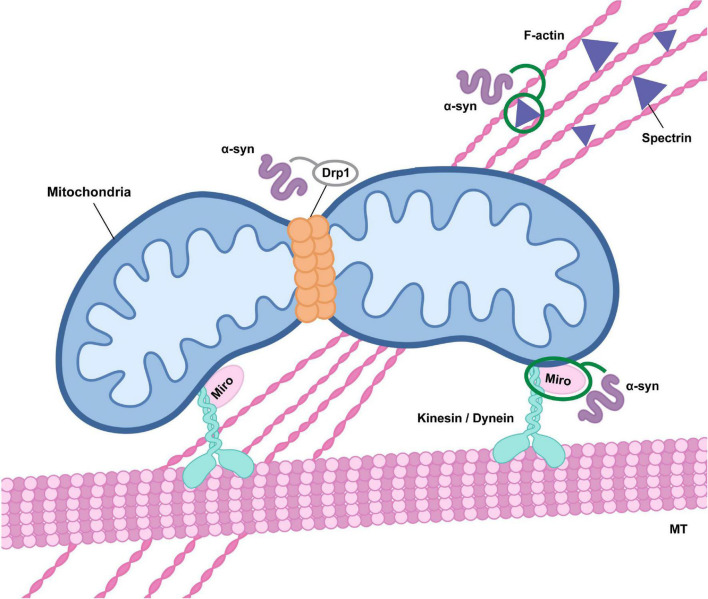
Alpha-synuclein influences mitochondrial transport and fission. Alterations in alpha-synuclein (α-syn) function may affect mitochondrial fission through direct effects on Drp1 activity and mitochondrial translocation, although the precise impact has not been clearly defined (indicated in gray). Oligomeric alpha-synuclein may also inhibit Drp1 trafficking to mitochondria as a result of alterations in actin cytoskeletal dynamics mediated by an association with the actin-cross linker spectrin. In addition, alpha-synuclein oligomers modulate Miro activity, either through promotion of Miro protein stability or retention in the outer mitochondrial membrane, influencing microtubule (MT) transport via dysregulation of kinesin or dynein activity.

Alpha-synuclein’s ability to associate with and remodel phospholipid membranes is a key feature that enables its physiological function at the synapse. In addition, it can interact with mitochondrial membranes, suggesting a role for alpha-synuclein in mitochondrial function and quality control. Membrane remodeling is a critical process required for the sequestration of damaged mitochondrial cargo in terms of both MDV formation and mitophagy, while also an essential mechanism to facilitate mitochondrial dynamics. Excess alpha-synuclein influences mitochondrial fission in both animal models and mammalian cells, and a recent study explored the mechanisms potentiating this using specific protein domain mutants of alpha-synuclein (Kamp et al., 2010; Nakamura et al., 2011; Butler et al., 2012; Furlong et al., 2020; Krzystek et al., 2021). Using a humanized Drosophila model, overexpression of full-length alpha-synuclein led to mitochondrial fragmentation which persisted in the absence of both the C-terminus and NAC domain, demonstrating the response was independent of alpha-synuclein’s propensity to aggregate (Krzystek et al., 2021). Fragmentation instead required an intact N-terminus, implying that the response was likely due to alterations in the biophysical properties of mitochondrial membranes resulting from alpha-synuclein interactions (Krzystek et al., 2021). Reduction of the essential mitochondrial fission machinery Drp1 in the context of alpha-synuclein overexpression resulted in a complete rescue of mitochondrial morphology, suggesting that alpha-synuclein-evoked fragmentation was dependent on Drp1 activity (Krzystek et al., 2021). Overexpression of the fusion protein Mfn2 did not evoke the same rescue, confirming the fragmentation response was a result of an increase in mitochondrial fission, rather than a decrease in mitochondrial fusion. Mitochondrial fission is thought to be initiated by recruitment of Drp1 to the OMM and previous studies have shown the translocation of Drp1 to mitochondria is significantly increased upon alpha-synuclein overexpression (Gui et al., 2012; Youle and van der Bliek, 2012). These data suggest a functional relationship between alpha-synuclein and Drp1 that could alter mitochondrial dynamics (Figure 1). Interestingly, this contradicts previous work which indicated that alpha-synuclein-induced mitochondrial fragmentation was completely independent of Drp1 (Nakamura et al., 2011). In this context, loss of Drp1 was not sufficient to rescue the fragmentation response following transient overexpression of alpha-synuclein. It was instead suggested that direct association of alpha-synuclein to the mitochondrial membrane was driving the fragmentation, since the response was abolished with overexpression of the A30P mutant of alpha-synuclein which lacks the ability to associate with membrane (Jo et al., 2002; Nakamura et al., 2011). Intermediate oligomeric alpha-synuclein species were also shown to directly fragment artificial phospholipid membranes *in vitro*, supporting alpha-synuclein’s potential as a direct modulator of membrane dynamics (Nakamura et al., 2011). In addition, fragmentation was not observed with alpha-synuclein monomers, mature oligomers or fibrils, suggesting that specifically intermediate, smaller oligomeric species were responsible for this effect (Nakamura et al., 2011). Though there is a consensus that alpha-synuclein overexpression can stimulate mitochondrial fission, there are still clear discrepancies about the mechanisms that drive this. One consideration is that alpha-synuclein may preferentially influence fission through an interaction with Drp1 when it is available, but in the absence of Drp1 it may or may not be able to stimulate fragmentation alone depending on its expression level and protein conformation.

**FIGURE 2:**
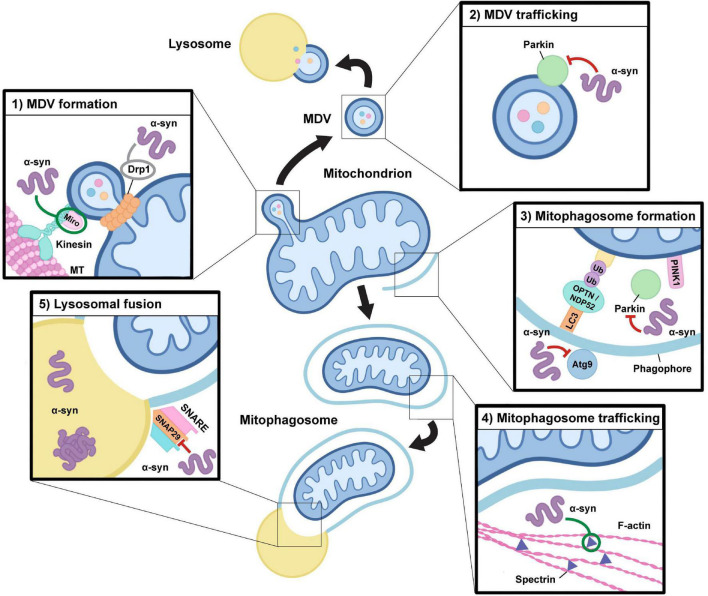
Alterations in alpha-synuclein function may impact mitochondrial quality control pathways. Alpha-synuclein function and its aggregation may have an impact at multiple levels during both mitophagy and the mitochondrial-derived vesicle pathway. (1) MDV formation: Drp1 and Miro proteins are required for mitochondrial-derived vesicle (MDV) fission from the mitochondrion in response to local oxidative damage, which may be directly influenced by alterations in alpha-synuclein (α-syn) function. Alpha-synuclein oligomers can stabilize Miro on the mitochondrial membrane and modulate Drp1 localization, although the precise impact of alpha-synuclein-induced alterations in Drp1 function is still a point of contention (indicated in gray). (2) MDV trafficking: Oligomeric species of alpha-synuclein may downregulate Parkin expression and alter its localization, which could have negative impacts on MDV formation and trafficking to the lysosome. (3) Mitophagosome formation: Mitophagy requires the action of PINK1 and Parkin to trigger the ubiquitylation of outer membrane proteins which leads to the recruitment of autophagy receptors, including NDP52 and OPTN, which facilitate the capture of damaged mitochondria within a phagophore, which matures into a mitophagosome. Alpha-synuclein may impact this process through alterations in Parkin activity and by inhibiting the recruitment of Atg9-positive vesicles which are required for autophagosomal membrane expansion. (4) Mitophagosome trafficking: Through interactions with spectrin, overexpression and accumulation of alpha-synuclein oligomers alters actin cytoskeletal dynamics resulting in its aberrant stabilization, which may negatively impact the maturation and trafficking of mitophagosomes required for endosomal and lysosomal fusion. (5) Lysosomal fusion: To enable cargo degradation, the mitophagosome requires the action of SNARE protein complexes to facilitate lysosomal fusion. Pathogenic overexpression of alpha-synuclein may alter SNAP29 activity, thus influencing the ability of mitophagosomes to fuse with lysosomes. In addition, accumulation of monomeric and oligomeric species of alpha-synuclein within lysosomes alters their activity, which may result in negative impacts on cargo degradation in both the mitophagy and MDV pathways.

Conversely, several studies report an enlargement in mitochondria in models of alpha-synuclein pathology, correlating with a decrease in Drp1 translocation from the cytoplasm to mitochondria (Ordonez et al., 2018; Portz and Lee, 2021). Pathogenic alpha-synuclein overexpression has been observed to decrease mitochondrial fission, which has been suggested to be due to abnormal stabilization of the actin cytoskeleton via an association with spectrin, thus preventing the trafficking of Drp1 to mitochondria (Figure 1; Korobova et al., 2013; Ordonez et al., 2018). In a human alpha-synuclein transgenic Drosophila model, a reduction in mitochondrial localization of Drp1 and subsequent decrease in fission could be rescued by genetic manipulation of actin (Ordonez et al., 2018). This mechanism of cytoskeletal modification by aggregate-prone proteins has previously been described in the context of other neurodegenerative diseases such as AD, where microtubule destabilization by hyperphosphorylated Tau drives the protein’s toxic effects both on mitochondria and protein trafficking (DuBoff et al., 2012). For alpha-synuclein, interactions with the actin cross-linking protein spectrin subsequently disrupts the spectrin organization and alters actin cytoskeletal dynamics (Machnicka et al., 2012; Ordonez et al., 2018). However, modification of the actin cytoskeleton not only impacts on mitochondrial dynamics, but can also directly impact on mitophagy (Sarkar et al., 2021). Specifically, pathogenic alpha-synuclein-induced actin stabilization has been shown to disrupt autophagosome trafficking to the lysosome, resulting in impaired autophagosome maturation (Figure 2; Sarkar et al., 2021). By the same reckoning, there is a potential for alterations in the actin cytoskeleton to disrupt trafficking of cellular components on a global scale, which would include MDVs and other endolysosomal compartments. As such, alpha-synuclein-induced modification of the actin cytoskeleton could have widespread cellular consequences (Oliveira da Silva and Liz, 2020). In terms of Drp1, recent work has determined it to be essential for the scission of MDVs from the mitochondria (König et al., 2021), so any disruption in Drp1 activity would alter MDV formation. More globally, Drp1 has defined roles in autophagy, apoptosis and cytoskeletal remodeling, so alpha-synuclein-induced alterations in its function could have broad cellular impacts (Frank et al., 2001; Duan et al., 2020). Reflecting the general tone of the literature, the majority of research has focused on overexpression models to assess the impact of alpha-synuclein on mitochondrial dynamics. However, one study investigating the effects of loss of function found that alpha-synuclein null mice had no change in Drp1 levels, suggesting that many of the impacts may be due to pathological forms of alpha-synuclein, primarily oligomeric species (Faustini et al., 2019). Supporting this, mitochondrial accumulation of alpha-synuclein is increased with the mutant A53T form (Devi et al., 2008). Since the A53T mutant has higher aggregation propensity, this suggests that aggregation and oligomerization could significantly alter mitochondrial dynamics.

## Alpha-synuclein and mitochondrial quality control

The function of essential mitophagy regulators PINK1 and Parkin are directly linked to alpha-synuclein-induced mitochondrial alterations. Parkin is functionally associated with several aspects of mito-QC distinct from mitophagy, such as the UPS and more recently in the MDV pathway where it has been shown to mediate both formation and trafficking of different classes of MDVs, essential for efficient cargo degradation by the lysosome (Shimura et al., 2000; McLelland et al., 2014; Ryan et al., 2020). In a neuronal cell model, exposure to exogenous alpha-synuclein oligomers or fibrils led to a reduction in Parkin expression alongside loss of mitochondrial membrane potential, decreased ATP production and increased mitochondrial ROS levels (Wilkaniec et al., 2019, 2021). Further assessment revealed alterations in mitophagy, exhibited by a reduction in mitochondrial protein ubiquitylation and subsequently less mitochondria present within autophagosomes (Wilkaniec et al., 2021). These mitochondrial phenotypes could all be rescued by Parkin overexpression, suggesting that an alpha-synuclein-induced downregulation of Parkin was responsible for mitochondrial dysfunction (Figure 2; Wilkaniec et al., 2021). Previous work has indicated addition of exogenous alpha-synuclein oligomers induces oxidative and nitrosative stress resulting in post-translational modifications to Parkin. In particular, *S*-nitrosylation of Parkin results in its autoubiquitination and degradation (Yao et al., 2004; Kazmierczak et al., 2008; Wilkaniec et al., 2019). This suggests a mechanism by which pathogenic alpha-synuclein can evoke Parkin downregulation, resulting in a damaging feedback loop that exacerbates mitochondrial damage due to loss of Parkin’s protective role against alpha-synuclein toxicity (Jêśko et al., 2019). Several studies report the ability of Parkin to restore mitochondrial morphology and function following alpha-synuclein-induced alterations, but it is unclear whether this is through a direct association between Parkin and alpha-synuclein, or more generally due to its neuroprotective role in regulating mitochondrial protein degradation (Kamp et al., 2010; Lonskaya et al., 2013; Jêśko et al., 2019; Krzystek et al., 2021). A functional relationship between PINK1/Parkin and alpha-synuclein has been suggested, exhibited by rescue of mitochondrial fragmentation and dysfunction by PINK1/Parkin overexpression, which is dependent on the C-terminus of alpha-synuclein (Krzystek et al., 2021). Furthermore, PINK1 and Parkin expression prevented alpha-synuclein-induced mitochondrial depolarization and neuronal death (Krzystek et al., 2021). Calcium binds alpha-synuclein’s C-terminus, which inherently increases its lipid-binding capacity, suggesting the association between PINK1/Parkin and alpha-synuclein requires membrane interactions (Lautenschläger et al., 2018; Krzystek et al., 2021). Alternatively, PINK1 has been shown to form a complex with alpha-synuclein in the cytoplasm and initiate autophagy to remove excess alpha-synuclein, potentially providing a protective mechanism against pathogenic forms of alpha-synuclein (Liu et al., 2017).

Alpha-synuclein may also influence mitophagy independently of PINK1/Parkin activity, instead through an interaction with Miro proteins, which are essential components of the machinery required for mitochondrial motility (Figure 1; Shaltouki et al., 2018). Functional mitochondria require Miro on their OMM to facilitate movement along microtubules, but it must be promptly degraded upon mitochondrial damage to halt motility and enable the initiation of mitophagy (Hsieh et al., 2016). Miro expression has been shown to be increased in PD brains post-mortem relative to healthy controls, and data from human neurons and a Drosophila model overexpressing alpha-synuclein also revealed an increase in Miro expression (Shaltouki et al., 2018). PINK1 and Parkin expression and mitochondrial recruitment in these models remains unchanged, suggesting the delay in mitophagy observed is likely the result of a direct alpha-synuclein-induced increase in Miro (Shaltouki et al., 2018). In addition, Miro reduction was able to rescue the delay in mitophagy and prevent degeneration of dopamine neurons in a Drosophila model expressing human alpha-synuclein, confirming the defect was attributed to Miro dysregulation (Shaltouki et al., 2018). Alpha-synuclein did not evoke alterations in Miro mRNA expression but instead was incorporated in the membrane-bound Miro complex, so could either be acting to stabilize Miro or prevent Miro removal from the OMM (Wang et al., 2011; Shaltouki et al., 2018). Using skin fibroblasts from PD patients, a recent study found that more than 94% of patient cell lines were unable to extract Miro1 from the OMM following depolarization, indicating that defects in Miro removal could be the mechanism driving the alpha-synuclein-induced delay in mitophagy observed in animal and cellular models (Hsieh et al., 2019). Intriguingly, Miro proteins have also been implicated in MDV biogenesis, with super-resolution microscopy revealing that mitochondrial membrane protrusions extend from the main organelle using microtubule filaments dependent on Miro1/2 activity, prior to Drp1-mediated scission (König et al., 2021). Alpha-synuclein-induced Miro stabilization therefore has the potential to impact multiple mito-QC mechanisms, which includes those driven by MDVs (Figure 2). A better understanding of the precise mechanisms of MDV formation and trafficking is needed to fully delineate these relationships. Since many of these studies have looked at either overexpression of alpha-synuclein or its pathological forms, it would be valuable to define the relationship between Miro proteins and endogenous alpha-synuclein. Could these interactions have a physiological function in the OMM-bound Miro complex and play a role in the induction of mitophagy?

Pathogenic overexpression of alpha-synuclein elicits alterations in autophagosome formation, maturation and autophagosome-lysosome fusion (Winslow et al., 2010; Sarkar et al., 2021; Tang et al., 2021). This has been characterized by reductions in the autophagosome associated LC3-II species and accumulation of known autophagy substrates, such as pathogenic forms of Huntingtin polyQ protein (Winslow et al., 2010). Previous research has implicated a protective role for the small GTPase Rab1 against alpha-synuclein toxicity in the context of ER-Golgi vesicular trafficking and autophagy (Cooper et al., 2006; Winslow et al., 2010). Supporting this, knockdown of Rab1a mirrored the effects observed following alpha-synuclein overexpression, suggesting that alpha-synuclein could be evoking autophagic dysregulation through alterations in Rab1a activity (Winslow et al., 2010). Furthermore, the alpha-synuclein induced reduction in autophagosomes could be rescued with Rab1a overexpression, insinuating that the two proteins were acting at similar stages of the autophagy pathway (Winslow et al., 2010). This was suggested to be early during autophagosome formation, based on the disruption of Atg9 localization to LC3-positive compartments; a step which is known to facilitate the delivery of membrane required for autophagosome expansion (Figure 2; Feng and Klionsky, 2017). Interestingly, defects in autophagosome formation were specific to wild-type alpha-synuclein in this model and were not observed with the A53T or A30P missense mutants (Winslow et al., 2010). Since these point mutations exist within the N-terminus of alpha-synuclein, their lack of influence on autophagosome formation may be partially due to altered membrane binding properties (Jo et al., 2000, 2002).

Several studies have also indicated that excess alpha-synuclein may impair autophagy further downstream, illustrated by a decrease in autophagic turnover as a result of defective autophagosome-lysosome fusion (Figure 2; Sarkar et al., 2021; Tang et al., 2021). This fusion process is mediated by a SNARE complex comprising Syntaxin17, SNAP29 and VAMP8 or YKT6, tethering together the two compartments to facilitate autophagosome maturation (Itakura et al., 2012; Guo et al., 2014; Matsui et al., 2018). Alpha-synuclein is known to promote the assembly of SNARE complexes at the synapse as part of its physiological function in neurotransmitter release (Burré et al., 2010). As such, alpha-synuclein’s interaction with SNARE complexes in the context of autophagosome-lysosome fusion was investigated to delineate potential mechanisms driving defective fusion (Tang et al., 2021). SNAP29 was found to be significantly less abundant in the context of alpha-synuclein overexpression, and subsequent co-expression of SNAP29 with alpha-synuclein restored autophagic flux (Tang et al., 2021). Interestingly, a reduction in SNAP29 was also seen in human SNpc neurons from patients with Lewy-body pathology, supporting a potential role for alpha-synuclein-induced dysfunction in the autophagic SNARE complex (Tang et al., 2021). Though the impact of alpha-synuclein has been studied in terms of overexpression, there is unexplored potential for endogenous alpha-synuclein to be playing a physiological role in mediating SNARE complex formation during autophagy as it does at the synapse. It could also be that alpha-synuclein only induces autophagy dysfunction above a certain threshold, which may not only depend on its expression level but also its conformation. Autophagy is essential for the removal of aggregation-prone proteins such as alpha-synuclein, so disruption of autophagosome formation and autophagosome-lysosome fusion by pathogenic forms of alpha-synuclein would generate a destructive feedback loop, potentiating the pathology (Gidalevitz et al., 2006; Ebrahimi-Fakhari et al., 2011; Tang et al., 2021).

## Conclusion

Alpha-synuclein and mitochondrial dysfunction have both been established as clear drivers of PD pathology, with evidence from PD patients and animal models confirming a relationship between the two (Ellis et al., 2005; Devi et al., 2008; Chinta et al., 2010; Ludtmann et al., 2016). The precise mechanisms behind this association are still unclear, but research is beginning to extrapolate roles for alpha-synuclein in mitochondrial dynamics and mito-QC. These associations are underpinned by alpha-synuclein’s ability to bind and remodel phospholipid membranes and interact with key signaling molecules involved in mitochondrial health and homeostasis.

Alpha-synuclein’s ability to directly bind the OMM, IMM, and TOM complexes on mitochondria demonstrates its potential to influence protein import as well as mito-QC systems that rely on membrane remodeling (Devi et al., 2008; Di Maio et al., 2016). Alpha-synuclein’s relationship with proteins such as Drp1, PINK1, Parkin, and Miro have confirmed its potential to impact quality control pathways such as mitophagy (Shaltouki et al., 2018; Wilkaniec et al., 2019; Krzystek et al., 2021). Additionally, the association of alpha-synuclein with Rabs and SNARE proteins at the autophagosome suggests the potential for alpha-synuclein-induced alterations at many stages of the PINK1/Parkin dependent mitophagic pathway (Sarkar et al., 2021; Tang et al., 2021). These mitochondrial protein interactions have been contextualized within mitophagy, but the MDV pathway utilizes similar machinery, such as the key regulators Miro1/2, Drp1, and Parkin. Accordingly, pathogenic forms of alpha-synuclein have the potential to influence MDV biogenesis and trafficking, though this remains unexplored.

Though much research has revealed the ability of alpha-synuclein to influence mitochondrial dynamics and mito-QC, studies have mostly used pathogenic overexpression models or the use of exogenous pre-formed alpha-synuclein fibrils to recapitulate PD pathology. Additional investigation into the physiological role of endogenous alpha-synuclein in these processes would be valuable to help to define its function away from the synapse and inform research on PD. Likewise, delineation of the precise mechanisms regulating mito-QC processes and defining how cargo selectivity is determined will help to build an understanding of the impact of mito-QC dysfunction on PD and its relationship with alpha-synuclein function and pathology.

## Author contributions

NT performed the research, produced the figures, and wrote the manuscript. DT led the study and edited the manuscript. Both authors contributed to the article and approved the submitted version.
